# A corpus of Chinese word segmentation agreement

**DOI:** 10.3758/s13428-024-02528-8

**Published:** 2024-12-28

**Authors:** Yiu-Kei Tsang, Ming Yan, Jinger Pan, Megan Yin Kan Chan

**Affiliations:** 1 Department of Education Studies, Hong Kong Baptist University, Kowloon Tong, Kowloon, Hong Kong; 2 Centre for Learning Sciences, Hong Kong Baptist University, Kowloon, Hong Kong; 3 Department of Psychology, University of Macau, Macau, China; 4 Center for Cognitive and Brain Sciences, University of Macau, Macau, China; 5 Department of Psychology, The Education University of Hong Kong, Ting Kok, Hong Kong

**Keywords:** Chinese reading, Word segmentation, Word boundary agreement, Corpus, Eye tracking

## Abstract

The absence of explicit word boundaries is a distinctive characteristic of Chinese script, setting it apart from most alphabetic scripts, leading to word boundary disagreement among readers. Previous studies have examined how this feature may influence reading performance. However, further investigations are required to generate more ecologically valid and generalizable findings. In order to advance our understanding of the impact of word boundaries in Chinese reading, we introduce the Chinese Word Segmentation Agreement (CWSA) corpus. This corpus consists of 500 sentences, comprising 9813 character tokens and 1590 character types, and provides data on word segmentation agreement at each character position. The data revealed a high level of overall segmentation agreement (92%). However, participants disagreed on the position of word boundaries in 8.96% of the cases. Moreover, about 85% of the sentences contained at least one ambiguous word boundary. The character strings with high levels of disagreement were tentatively classified into three categories, namely the morphosyntactic type (e.g., “反映–了”), modifier–head type (e.g., “科學–教育”), and others (e.g., “大力–支持”). Finally, the agreement scores also significantly influenced reading behaviors, as evidenced by analyses with published eye movement data. Specifically, a high level of disagreement was associated with longer single fixation durations. We discuss the implications of these results and highlight how the CWSA corpus can facilitate future research on word segmentation in Chinese reading.

## Introduction

Much research in psycholinguistics, including the exploration of reading processes through eye tracking, has been predominately conducted in English and other alphabetic scripts. Nonetheless, considering the diverse range of writing systems globally, it is crucial to examine various scripts to comprehend both script-specific and universal aspects of reading processes. In the past two decades, Chinese has emerged as a significant script for eye movement research in reading, second only to English (Siegelman et al., 2022). Adopting a logographic writing system, Chinese possesses unique features that set it apart from alphabetic scripts, making it an ideal candidate for meaningful comparisons between scripts.

**Fig. 1:**
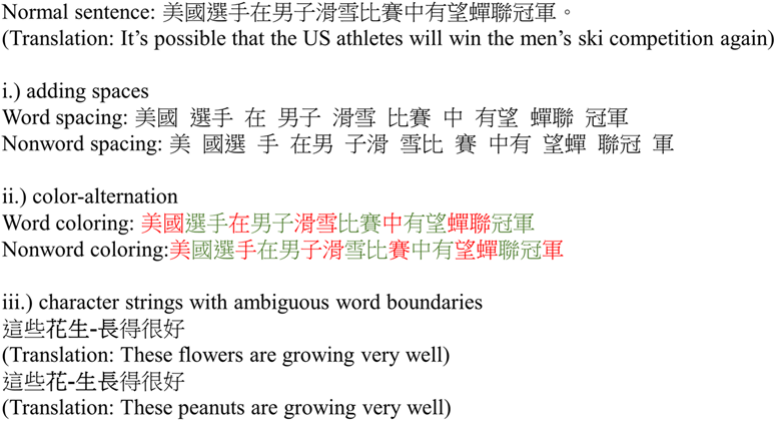
Some common manipulations for testing the influence of the absence of explicit word boundaries on eye movement control in Chinese sentence reading, including **i**.) adding spaces that aligned or misaligned with word boundaries, **ii.**) using color alternation that aligned or misaligned with word boundaries, and **iii.**) using character strings with ambiguous word boundaries. Importantly, these manipulation involve unusual presentation or materials, potentially compromising the generalizability and external validity of the findings

A particularly distinct trait of Chinese script is the absence of explicit word boundaries, which can have profound implications for eye movement control during reading (Tsang & Chen, 2012). Previous studies exploring this aspect have often relied on some unnatural manipulations (Fig. 1), such as inserting spaces to denote word boundaries (Hsu & Huang, 2000a, b) or highlighting word boundaries with colors (Perea & Wang, 2017; Zhou et al., 2018). Another approach involves using character strings with ambiguous boundaries (Inhoff & Wu, 2005; Yan & Kliegl, 2016), such as the example “花生長,” which can be segmented as 花–flower 生長–to grow” or “花生–peanut 長–to grow.” However, it is important to recognize that the issue of word boundary ambiguity extends to all Chinese words, rather than being limited to these special cases (Hoosain, 1992). Thus, relying solely on these manipulations to draw conclusions may pose threats to ecological validity and generalizability. The primary objective of this study is to address this gap by presenting the Chinese Word Segmentation Agreement (CWSA) corpus. This corpus comprises segmentation agreement ratings for 500 Chinese sentences, totaling 9813 character tokens and 1590 unique character types (i.e., a token-to-type ratio of 6.17:1), obtained from 80 native Chinese speakers. Notably, these sentences were extracted from different sources (e.g., news articles, supplement articles of online newspaper, encyclopedia entries, and a novel), ensuring that they are representative of natural language usage. By utilizing this corpus and segmentation agreement scores, researchers can investigate word boundaries in Chinese reading with more ecologically valid and generalizable materials.

In the following, we elaborate on how the absence of word boundaries may impact Chinese reading, and we review previous studies that have examined this topic. We then describe the development of the CWSA corpus and how we validate the segmentation agreement scores obtained. Specifically, using the data from the Beijing Sentence Corpus (BSC; Pan et al., 2022a; Yan et al., 2010) and three published studies (Pan et al., 2022b, 2023; Yan et al., 2023), we demonstrate that after controlling for standard linguistic variables (e.g., word frequency and word length), these scores can still significantly affect eye movement behaviors during Chinese sentence reading. Finally, we discuss how the CWSA corpus can be utilized in future research to advance our understanding of Chinese reading processes.

### Lack of explicit word boundaries in Chinese

In English and many alphabetic scripts, words within sentences are separated by physical spaces. These spaces are useful for reading because they allow readers to easily recognize word boundaries and target their fixations near the center of each word for better word recognition efficiency (McConkie et al., 1989; O’Regan et al., 1984). As a consequence, first-fixation location typically forms a Gaussian distribution with its peak around the word center, known as the preferred viewing location (PVL; Rayner, 1979). In fact, studies have shown that removing spaces between words in English significantly impairs reading speed and oculomotor behaviors (Perea & Acha, 2009; Rayner et al., 1998). However, as shown in Fig. 1, Chinese script naturally lacks spatial cues that explicitly mark word boundaries. This raises the intriguing question: How can it be that reading efficiency in Chinese remains comparable to that of alphabetic scripts (Brysbaert, 2019), despite the absence of word boundary cues?

One simple explanation is that in Chinese reading, the basic unit guiding eye movement control is the individual character rather than the word (Chen et al., 2003). Given that Chinese characters are often semantically ambiguous while words are not, it has been argued that word recognition is more efficient when the word is accessed holistically (e.g., Packard, 1999). However, previous research in isolated word processing has shown that even when Chinese words contain ambiguous characters, these characters still play a role in the word recognition process (Tsang & Chen, 2013a, b). Although to the best of our knowledge, there is no direct evidence that morphological ambiguity affects where the eyes move during reading, it is possible that characters play a more important role than words in guiding fixations in reading. Alternatively, Chinese readers may adopt a fixed-length saccade strategy with some random errors (e.g., Li et al., 2011; Yang & McConkie, 1999; see also Yan et al., 2010, p. 717, for a simulation of fixed-amplitude saccade). In particular, Tsai and McConkie (2003) observed a flat distribution of first-fixation location and thus reported no evidence for PVL in Chinese. They concluded that, unlike other spaced scripts, saccade generation in Chinese may not be word-based. That is to say, both models claim that word segmentation is simply not an essential process in Chinese reading, and the absence of explicit word boundary cues is not a problem at all. Indeed, some researchers proposed a processing-based saccade targeting mechanism in Chinese (Liu et al., 2015; Xia et al., 2023) which assumes that during each fixation, readers attempt to process as many characters as possible and program their saccades to the next unprocessed character. Then, saccade targeting is mainly determined by factors such as an individual’s processing capacity and information load at the current fixation point. The salience of word boundaries only indirectly affects outgoing saccade amplitude through modulating the foveal processing load.

On the other hand, it is well established that word properties like frequency and length can influence eye movement behaviors in Chinese reading (e.g., Li et al., 2014; Pan et al., 2022a). In a direct comparison between the effects of character and word frequency in Chinese reading, Li et al. (2014) indeed demonstrated that word frequency showed stronger influences than did character frequency on various eye movement indices, including gaze durations and forward saccade lengths. To accurately describe the word effects in Chinese reading, it is essential for reading models to incorporate mechanisms that explain how word segmentation can be done successfully without relying on spatial cues.

Yan and colleagues proposed a flexible mechanism which dynamically incorporates both word-based and character-based saccade targeting. Firstly, word segmentation during Chinese reading is performed using parafoveal vision (Yan & Kliegl, 2016; Yan et al., 2010). Given that Chinese readers have a perceptual span covering three to four characters along the reading direction (Inhoff & Liu, 1998; Yan et al., 2015), and considering that around 70% of Chinese word types consist of two characters and over 90% range from one to four characters long (Tsang et al., 2018), it can be inferred that the parafoveal region contains approximately two upcoming words. This enables the cognitive system to utilize the information in the parafovea, such as transitional probability between characters (Yen et al., 2012), to identify word boundaries prior to moving the eyes to the next word for lexical access. Consequently, the location of the next fixation will be sensitive to properties of the upcoming word and is likely to be near the word’s center for better word recognition efficiency—similar to what occurs in alphabetic languages. Secondly, when parafoveal word segmentation is unsuccessful, readers will fixate at the first character of the next word. Recently, Fan and Reilly (2022) provided further support to the flexible saccade model. Focusing on typically developing readers, the authors proposed character-based eye guidance for long saccades and word-based eye guidance for short saccades, agreeing with Yan et al.’s assumption that word segmentation is more likely to fail when a saccade is launched farther away. Zang et al. (2018) also demonstrated that word length affected saccade target selection in Chinese reading. Specifically, when the upcoming word was three characters long, readers would make longer saccades to bring the eyes near the word center than when the upcoming word was just one character long.

### Previous studies on the role of word boundaries in Chinese reading

Researchers have been interested in examining how the absence of explicit word boundaries influences Chinese reading. As mentioned earlier, previous studies have adopted various approaches and manipulations. One research direction seeks to demonstrate that, similar to reading alphabetic scripts, a word serves as the fundamental unit in Chinese reading. Thus, the absence of explicit word boundaries in Chinese will hinder word segmentation and reduce reading efficiency. Conversely, when word boundary cues are provided, reading efficiency can be improved. In two early studies, Hsu and Huang (2000a, b) showed that inserting inter-word spaces (Fig. 1, top panel) increased overall reading speed for difficult sentences and sentences containing ambiguous word boundaries. In an eye-tracking study, Bai et al. (2008) observed that sentences with spacing misaligned with word boundaries were read more slowly than word-spaced sentences and normal unspaced sentences, although no benefits were found for word-spaced sentences. Together, these findings suggest that providing appropriate word segmentation cues may be beneficial in Chinese reading.

A notable limitation of the spacing manipulation used in previous studies is that it alters the physical layout of the sentence, essentially lengthening it. This led other researchers to employ color alternation as a means of providing boundary cues (Fig. 1, middle panel). For example, Perea and Wang (2017) observed a benefit on overall reading speed when word boundaries were marked by color, although the effect was limited to developing readers or when adults read technical text. In an eye-tracking study, Zhou et al. (2018) demonstrated that when the color marking corresponded with word boundaries, readers were more likely to fixate near the word center and had shorter gaze durations (i.e., the cumulative duration of all fixations during the first-pass reading of the word) compared to the uniform color baseline condition. Conversely, when the color marking did not align with word boundaries, the pattern was reversed, leading to fixation locations near the word’s beginning and longer gaze durations. Similar results were also observed in studies involving second language learners of Chinese (Zhou et al. 2020) and third-grade readers (Pan et al., 2021). These findings, which indicate that providing word boundary cues can enhance reading efficiency, agree well with the aforementioned proposal of parafoveal word segmentation in Chinese (Yan et al., 2010). When readers perceive visual cues about word boundaries in their parafoveal vision, they can utilize the information to guide their next fixation towards the word’s center for optimal processing.

Another area of research regarding word segmentation in Chinese focuses on how ambiguity in word boundaries affects the reading process. As depicted in Fig. 1 (bottom panel), approximately 3.6% of character strings (based on an analysis of the Academia Sinica balanced corpus reported in Yen et al., 2012) can be segmented into words in multiple ways, leading to entirely different meanings. Employing the eye-tracking technique, Inhoff and Wu (2005) explored the processing of four-character strings with ambiguous word boundaries in semantically neutral sentences. Specifically, for the character string “ABCD,” the correct segmentation should be “AB” and “CD.” However, “BC” could also form a word, and such ambiguity resulted in longer gaze duration than character strings without ambiguous boundaries (e.g., “FG” was not a word for “EFGH”). Replicating and extending the original work by Inhoff and Wu (2005), Yan and Kliegl (2016) further demonstrated that such ambiguous strings in the parafovea affected saccade amplitude and first-fixation location, indicating a parafoveal word segmentation effect on saccade generation in Chinese.

### The present study

The studies discussed above have provided valuable insights into how the absence of word boundary cues in Chinese can impact the reading process. The available evidence suggests that reading efficiency is indeed hampered in such a circumstance, as providing explicit segmentation cues can improve reading performance (e.g., Zhou et al., 2018). However, it is worth noting that such manipulations are arguably unnatural and could potentially lead to strategic processing that is not typical to natural reading. The absence of word boundary cues can also occasionally result in uncertainty or ambiguity in how to correctly segment character strings, further slowing reading speed (e.g., Inhoff & Wu, 2005). However, the kind of boundary ambiguity tested in previous studies (i.e., “ABCD” cannot be unambiguously segmented into “AB” and “CD” because “BC” is also a word) is indeed a special case. Firstly, as previously mentioned, such cases are relatively rare, occurring in only 3.6% of character strings (Yen et al., 2012). Secondly, there is a correct answer in this case, as the sentence cannot be comprehended if the character string is segmented as “A,” “BC,” and “D,” even when “BC” is a real word. Thirdly, in practical language use, when proficient Chinese users compose a sentence containing this kind of ambiguity, they can easily clarify their intended meanings by inserting a single character at the appropriate word boundary without greatly affecting the sentence structure and meaning. For example, in the sentence “這些**花生長**得很好–These flowers/peanuts are growing very well” shown in Fig. 1 (bottom panel), disambiguation can be achieved by inserting the character “都–all” at the word boundary, resulting in “這些**花**都**生長**得很好–All these flowers are growing very well.”

Word boundary ambiguity is indeed a more common phenomenon in Chinese beyond those special cases examined in previous studies. It can be found in many daily life reading materials, even though an ordinary Chinese reader may not be aware of it. In an early study involving nine simple sentences and one short passage from a grade 10 textbook, Hoosain (1992) observed substantial disagreements in word segmentation, both within the group of participants and between the participants as ordinary readers and the researcher as an expert. Similarly, in a larger scale study involving 142 participants and 200 sentences from a modern Chinese corpus (Center for Chinese Linguistics PKU), Liu et al. (2013) discovered that the word boundaries identified by the participants were often inconsistent, and the agreement proportion can be even lower than 50% in some cases (e.g., 34% agreement after an adjective).

To advance our understanding regarding how word segmentation affects Chinese reading, it is important to investigate more natural and common cases. Instead of relying solely on expert judgment by researchers or national standards, we propose that it would be fruitful to let ordinary Chinese readers define word boundaries in sentences. This approach would allow for the computation of segmentation agreement scores based on empirical data, where a higher agreement score indicates less ambiguity in word boundaries. These scores can serve as a foundation for developing new experiments that examine how word segmentation influences Chinese reading, using more natural materials and procedures. With this aim in mind, we introduce the CWSA (Chinese Word Segmentation Agreement) corpus below, which comprises segmentation agreement scores for 500 sentences.

## Method

### Participants

Eighty adult readers were recruited. These participants were native speakers of Cantonese Chinese, had received formal education in Hong Kong since kindergarten, and used traditional Chinese characters. None of the participants reported any history of language-related disorders or education needs. Informed consent was obtained from all participants before the study. The study protocol was approved by the Research Ethics Committee at Hong Kong Baptist University.

### Materials

The CWSA corpus consists of a total of 500 sentences. These sentences come from several sources and are of different contents and genres (e.g., news, entertainment, scientific knowledge, and novel), which increases the representativeness of these sentences in resembling the daily reading experiences of an ordinary Chinese reader. Firstly, 91 sentences were taken from the Beijing Sentence Corpus (BSC; Pan et al. 2022a; Yan et al., 2010). These sentences were extracted from an official newspaper in mainland China (People’s Daily) and were originally written in simplified Chinese. They were converted into traditional Chinese using the conversion tool in Microsoft Office. Care was taken to ensure correct conversion of characters with a non-one-to-one correspondence. For instance, the simplified character “后” could correspond to “後–back” or “后–queen” in traditional Chinese. Some editing was also performed on a few vocabulary items to replace words less commonly used in Hong Kong with more common ones (e.g., “營房–camp” was replaced with “帳蓬–tent”). These edits were made while carefully preserving the meaning, length, and structure of the sentences (see Pan & Yan, 2024, for further details). Despite difference in the spoken language, the written Chinese is rather universal in different regions, except for some very minor vocabulary differences. Pan and Yan (2024) slightly edited the BSC sentences for Hong Kong participants and found good comprehension (90.5% accuracy). The reading speed (403 characters per minute) observed was also comparable to the meta-analysis by Brysbaert (2019; 390 characters per minute).

Secondly, 184 sentences in traditional Chinese were selected from several recent studies (Pan et al., 2022b, 2023; Yan et al., 2023). The original experiments had used either an error disruption paradigm (Doctor & Coltheart, 1980) or a gaze-contingent boundary paradigm (Rayner, 1975), where a critical character was replaced foveally or parafoveally with different types of substitutions or previews in some conditions. For the present purpose, only the unmanipulated, “correct” version of the sentences was used.

Thirdly, 74 additional sentences were adapted from the Ghent Eye‑tracking Corpus for Chinese–English bilinguals (GECO-CN; Sui et al., 2023), an eye-tracking megastudy that involved participants reading the simplified Chinese version of Agatha Christie's novel *The Mysterious Affair at Styles*. Since the source material was a novel, it contained many proper names (e.g., character names) and short sentences separated by punctuation marks (e.g., in dialogue). Only sentences that were reasonably long and did not contain proper names were selected for this study. In some cases, the sentences used were edited to remove proper names or combined from two short sentences. The 74 sentences selected were converted into traditional Chinese using the same procedure as the BSC sentences. While the novel was originally in simplified Chinese, three research assistants, who are native Hong Kong Chinese readers, ensured that the sentences selected are natural to participants in Hong Kong.

Fourthly, 25 sentences came from the Multilingual Eye-movement Corpus (MECO; Siegelman et al., 2022). At the time of preparing the materials, the Chinese version of the MECO passages was not available. Therefore, the first author translated the English version passages into traditional Chinese and selected 25 sentences from nine of the translated passages.^1^ Finally, the first author created 126 new sentences by adapting content from supplement articles published in several Hong Kong online newspapers (HK01; MingPao; SingTao). These articles covered a diverse range of topics, including entertainment-focused subjects like food and travel, as well as more information-oriented content like science and health.

In total, the stimulus set comprised 500 sentences. The average sentence length was 19.63 characters, with a range of 14 to 25 characters and a standard deviation of 2.66. Across all sentences, there were 9813 character tokens and 1590 unique character types.

### Procedure

Data collection was done in two phases. In phase 1, the 91 BSC sentences and 89 of the new sentences were divided into two lists, each containing 90 sentences. In phase 2, the remaining 320 sentences were divided into two lists, each containing 160 sentences. In each phase, 40 participants were randomly assigned to complete one list until there were 20 participants assigned to each list. Having 20 data points for each item is comparable to other recent norming studies in Hong Kong (Chan & Tse, 2024; Su et al., 2023).

While it is interesting to obtain response times for the segmentation process, the complexity of the processes involved (e.g., sentence reading, word recognition, and decision making) makes a single response time measure less meaningful. Furthermore, as illustrated below, the offline record is sufficient to inform the Chinese reading process. Therefore, the segmentation task was administered in a paper-and-pencil format. Participants were provided with hard copies of the sentences, which were printed on A4-size paper, with one sentence per line. Participants were instructed to use slashes to indicate the positions of word boundaries within each sentence. For instance, if the participant considered “AB” as a word in the four-character sequence “ABCD,” they would place a slash between “B” and “C” (i.e., “AB/CD”). Alternatively, if they considered “ABC” as a word, the slash would be placed after “C” (i.e., “ABC/D”). Participants were informed that there were no correct answers for the segmentation task and were reminded to perform the task based on their first interpretation. The task required approximately 30 minutes (phase 1) or 50 minutes (phase 2) to complete (including instruction, clarification, and debriefing).

## Analyses and results

### Coding word segmentation

**Fig. 2:**
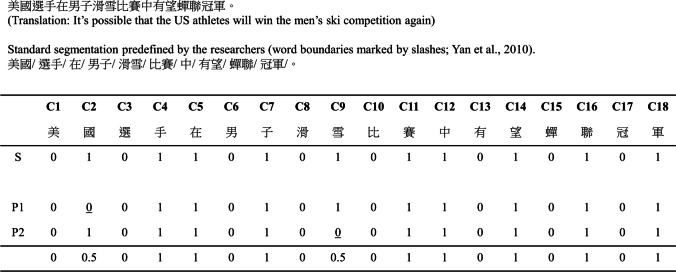
An illustration of the coding scheme, Following Yan et al. (2010), we used “0” to indicate “not a word boundary” and “1” indicate “a word boundary”, C1 to C18 refers to the character position in the sentence. The row “S” refer to the “standard” segmentation predefined by the researchers, The row “P1” and “P2” refer to the segmentation by the two hypothetical participants. The points of deviation from the standard are underlined. The bottom row is the agreement score reported in the present CWSE corpus

To quantify the word segmentation agreement among participants, a coding scheme similar to Yan et al. (2010, p. 725) was employed. In Yan et al.’s study, a binary code was used to indicate the presence or absence of a word boundary after each character in a sentence. A code of “0” represented “not a word boundary,” while a code of “1” represented “a word boundary.” Additionally, a predefined “standard” segmentation was established for each sentence, against which participants’ segmentations were compared. In this standard, a character string was considered a word if it had an entry in the *Modern Chinese word frequency dictionary* (Institute of Linguistic Studies, 1986). Figure 2 provides an example to illustrate this coding scheme. According to the standard segmentation for the sentence “美國選手在男子滑雪比賽中有望蟬聯冠軍” (It’s possible that the US athletes will win the men’s ski competition again), the word boundaries are as follows: 美國/ 選手/ 在/ 男子/ 滑雪/ 比賽/ 中/ 有望/ 蟬聯/ 冠軍/.” Converting this standard segmentation into a binary code sequence results in “010110101011010101.” Figure 2 also presents the segmentation provided by two hypothetical participants, P1 and P2. P1 deviated from the predefined standard by considering the four-character group “美國選手–US athletes” as one word instead of two. Similarly, P2 treated “滑雪比賽–ski competition” as one word instead of two. Both P1 and P2 differed from the standard by only one word boundary, indicating a high level of segmentation agreement with the standard. In this case, the agreement score is calculated as 17 correct word boundaries out of 18 total word boundaries, resulting in an overall agreement score of 17/18 = 0.94.

The coding scheme in Yan et al. (2010) requires a predefined standard segmentation, and the scoring system mainly provides an index of segmentation agreement at the global whole-sentence level. In contrast, the focus of the CWSA corpus was on agreement at the local level. Without predefining a standard segmentation for each sentence, we identified the characters on which participants disagreed regarding their status as word boundaries. For example, as shown in Fig. 2, both hypothetical participants, P1 and P2, agreed that C6 “男–male” was not a word boundary. Therefore, the segmentation agreement was 0/2 = 0, indicating a high degree of agreement that a word boundary should not be placed after this character. Similarly, the agreement score of C7 “子–son” was 2/2 = 1, indicating a high degree of agreement that there was a word boundary after this character. These scores together indicate a consensus among participants that the C6C7 two-character string “男子–men” was a word. On the other hand, disagreement was observed at C2 “國–nation” and C9 “雪–snow,” resulting in an agreement score of 1/2 = 0.5 at both positions. Given that the decision is binary, a score of 0.5 indicates a complete disagreement among participants and reflects the ambiguity regarding whether “美國選手–US athletes” or “滑雪比賽–ski competition” should be treated as one word or two words.

### Agreement in word segmentation

For each of the 500 sentences in the CWSA corpus, the segmentation agreement score was calculated at each character position using the segmentation data provided by the 20 participants. To obtain an index of overall segmentation agreement, raw values below 0.5 were reverse-scored, which converted 0 to 1, 0.1 to 0.9, and so on. The purpose of this conversion was to account for the fact that both 0 and 1 indicated perfect agreement among participants, albeit representing non-boundary and boundary, respectively. However, it is noteworthy that the range of the converted agreement score was 0.5 to 1 (while that of the original score was 0 to 1). After applying this conversion, the grand mean agreement score across the 9813 characters in the corpus was 0.92, with a standard deviation of 0.13. This high mean agreement score aligns with the overall high level of segmentation agreement reported in previous studies (Pan et al., 2022a, b; Yan et al., 2010). The value was also significantly higher than the baseline value of 0.5 (which indicates complete disagreement), as shown in a one-sample Student *t*-test (*t*(9813) = 320.04, *p* < .001).

Although there was a high level of global segmentation agreement in the CWSA sentences, instances of local disagreement were common. Out of the 9813 characters in the corpus, 879 (8.96%) had a converted agreement score between 0.5 to 0.7 (i.e., original agreement score between 0.3 to 0.7), indicating a lower level of consensus regarding the presence or absence of a word boundary at those positions. Furthermore, approximately 85% of the sentences in the corpus contained at least one character with a converted agreement score between 0.5 to 0.7, suggesting that ambiguous word boundaries were present in the majority of the sentences. If a more stringent criterion was applied, considering a converted agreement score between 0.5 to 0.8 as an indicator of low agreement, the number of characters with low agreement increased to 1439 (14.66%). In this case, 95% of the sentences contained at least one ambiguous word boundary.

Further analysis of the character strings with low agreement scores (0.3 to 0.7 in raw score, 0.5 to 0.7 in converted score) in the CWSA corpus revealed that ambiguity in segmentation could be classified into three main categories. The first category of ambiguity involved characters that functioned as affixes in derivational or inflectional morphology. Participants exhibited inconsistency in determining whether these affix-like characters should be considered independent one-character words or combined with the stems (which were usually two-character words) to form derived or infected words. For instance, there was disagreement among participants regarding the segmentation of “反映了–reflected,” which had a converted agreement score of 0.5 (i.e., complete disagreement) at “映.” Some participants considered it a single three-character word, while others segmented into two separate words “反映–to reflect” and “了–a past tense marker similar to -ed.” Similarly, participants disagreed on whether “不穩固–unstable” (converted agreement score = 0.6) should be treated as one word or two separate words: “不–a negation prefix similar to non-” and “穩固–stable.” Another example involves the character “的,” which can act as an adjective marker, as in “溫暖的–warm” (converted agreement score = 0.6), or act as a possessive marker, as in “我的–my” (converted agreement scores = 0.5). This type of ambiguity was also found in Liu et al. (2013), with examples such as the adverb marker “地” in “迅速地–quickly” and the position marker “在” in “躺在–to lie on.” Here, we labeled this type of ambiguity as the “morphosyntactic type,” totaling 243 cases.

The second type of ambiguity involved two multi-character nouns that could be used independently in other contexts. However, when one word immediately followed the other, participants disagreed on whether the combination should be considered as a single word with a modifier–head structure or should remain as two separate words. For example, “科學–science” and “教育–education” were both two-character words by themselves, but their combination “科學教育–science education” had a converted agreement score of 0.55 between the second and third characters, indicating high disagreement among participants. As another example, the combination “節日氣氛–festive atmosphere” could be constructed from “節日–festival” and “氣氛–atmosphere.” Its converted agreement score was 0.6. This type of ambiguity, which is analogous to the disagreement between “video game” and “videogame” in English, was labeled as the “modifier–head type,” comprising 301 cases. Hoosain (1992) reported a similar case for “自然科學–natural science.”

Finally, there were some idiosyncratic cases that did not fit well into the two mentioned categories. Some of them might be potentially classified into specific structures like coordinative compounds (e.g., “體育–sport” and “運動–exercise” could form “體育運動–sport,” which had a converted agreement score of 0.65). However, the number of these cases was too small to form a separate category. Other cases had diverse structures and could not be easily grouped. For example, both “大力支持–strongly support” and “奇蹟反勝–miraculous reversal” had a low converted agreement score of 0.65 between the second and third characters. These cases formed the “other type” group. Interested readers are encouraged to download CWSA for more examples in each category and to explore the possibility of alternative classifications.

### Validation with eye-tracking data

To validate the segmentation agreement data in the CWSA corpus, we tested how the agreement score influenced eye movement behaviors in Chinese sentence reading, using the data from BSC and other experiments (Pan et al., 2022a, b, 2023; Yan et al., 2010; 2023), which will henceforth be referred to as phase 1 and phase 2 data, respectively. We did not use the data from GECO-CN because of the more extensive editing on these sentences (see Materials section). Similarly, MECO data was not used because the Chinese version data was not made public at the time of analyses. The phase 1 and 2 data were analyzed separately because BSC contained only natural sentences without specific manipulations, while the other experiments involve experimental manipulations (although only the correct version was used in constructing CWSA). In addition, information about predictability was available for BSC but not for the other experiments.

The phase 1 data consisted of eye movement data from 60 undergraduate students in Beijing, who read 150 sentences in simplified Chinese. The sentences were segmented into words based on predefined standards. As mentioned before, a character string was taken as a word when it has an entry in the word frequency corpus by Institute of Linguistic Studies (1986). Using this criterion, the mean word length of the 91 sentences used in this study was 1.86 (*SD* = 0.54). Major eye movement variables provided by BSC include fixation location and duration on words, saccade amplitudes, saccade direction, and so on. It also provides several word-level linguistic variables, such as frequency, length, number of strokes, and predictability of the fixated words. Although we collected data with traditional Chinese participants in Hong Kong, word segmentation is more related to lexical knowledge than the visual form of the characters. Care was also taken to preserve the sentence structure and length when the materials were converted from simplified to traditional Chinese. In any case, the mismatch in script would probably reduce the explanatory power of the agreement scores in the CWSA corpus. If the agreement scores could nevertheless produce significant effects on eye movement behaviors, it would provide stronger evidence for the validity of the CWSA corpus. Therefore, for the present purpose of validating the segmentation agreement scores, the data should provide a reasonable approximation.

A low converted agreement score (i.e., near 0.5) indicated word boundary ambiguity. It is expected that such ambiguity would increase the difficulty of lexical processing, resulting in longer fixation durations. To test this prediction, we analyzed the eye movement data with linear mixed-effects model (LMM) implemented in the R platform (version 4.3.1; R Core Team, 2023), using the *lme4* package (Bates et al., 2015) for model fitting and the *lmerTest* package (Kuznetsova et al., 2017) for obtaining *p*-values based on the Satterthwaite approximation. The dependent variable was log-transformed single fixation duration (i.e., the duration of the fixation on a word when it is fixated upon exactly once during the first-pass reading). The majority of the data points (i.e., 83% for phase 1 data and 84% for phase 2 data) were single fixations. In contrast to multi-fixation cases, single-fixation cases carry few mislocated fixations and oculomotor errors (Nuthmann et al., 2005; Vitu et al., 2001) and are associated with more completed word segmentation in Chinese reading (e.g., Yan et al., 2010). In addition, the choice of using single fixation duration as the dependent variable makes for better comparison with some previous corpus analyses (e.g., Kliegl et al., 2006).

Fixed effects for the model included the word-level linguistic variables provided in BSC, namely (i) log-transformed word frequency, (ii) word length (reciprocal of number of characters), (iii) predictability (logit-transformed), and (iv) visual complexity in number of strokes of the word. These predictors were control variables. The predictor of interest, segmentation agreement (i.e., the converted score which ranges from 0.5 to 1), was further converted into a disagreement score (i.e., 1 minus the converted agreement score) for easy interpretation because the predictions were made with reference to disagreement. Moreover, given that the score was essentially a percentage, logit transformation was performed to account for range restriction. Following Cohen et al. (2003), zero disagreement values were replaced with 1/(2×20), where 20 represents the number of participants contributing to the original scores. The values of all predictors were standardized into *z*-scores to eliminate differences in measurement scale, which simplifies interpretation (i.e., the intercept is the grand mean average fixation duration) and comparison of the sizes of regression coefficients (i.e., changes per SD). Moreover, *z*-score transformation also centers the variables, reducing the problem of multi-collinearity (especially when higher order effects are tested; Cohen et al., 2003). For the random effect structure, we entered by-participant and by-sentence random intercepts, and by-participant random slopes of the predictors. In the cases in which the model failed to converge or a singular fit was obtained, we removed the random effect that had the smallest variance and reran the model. The process continued until the model converged successfully. The data and script for the analyses, and the detailed outputs can be found at https://osf.io/m3rcf/.

**Fig. 3:**
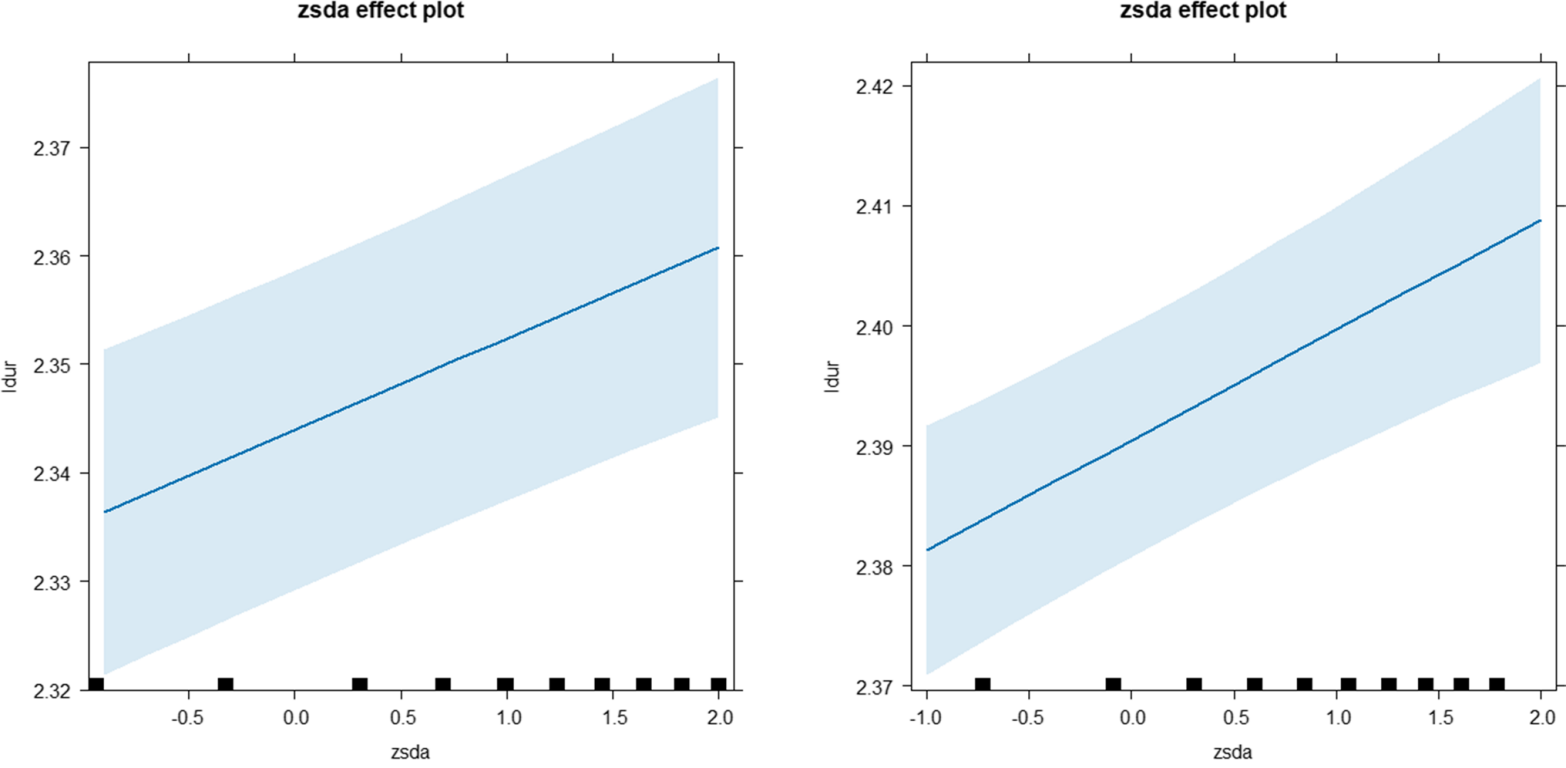
The partial relationship between z-score transformed segmentation disagreements and log-transformed single fixation duration in the BSC data (left panel) and phase 2 data (right panel). The 95% confidence intervals are displayed around the best-fit lines

Table 1 shows the result of the LMM model for phase 1 data. After accounting for the effects of several word-level linguistic variables, segmentation disagreement continued to significantly affect the log-transformed single fixation durations (*b* = 0.0071, *SE* = 0.00085, *t* = 8.28, *p* < .001) on the fixated word. Consistent with the predictions, ambiguity in word boundaries led to longer single fixation durations. Figure 3 (left panel) illustrates the partial relationship between the disagreement score and the log-transformed single fixation durations (i.e., the model estimates after statistically controlling for fixed and random variables) for the phase 1 data.

**Table 1:** LMM results on log-transformed single fixation durations of phase 1 (Beijing Sentence Corpus) sentences

	Estimates	Standard errors	*t* values
Intercept	2.34	0.0075	312.46*
Word frequency	−0.0089	0.0024	−3.75*
Word length	−0.012	0.0029	−4.18*
Predictability	−0.0037	0.0020	−1.79
Word number of strokes	0.013	0.0020	6.59*
Segmentation disagreement	0.0084	0.0015	5.81*

* *p* < .001

The same analysis was conducted on the sentences in phase 2 data, except that predictability information was unavailable for these sentences and was thus excluded from the model. The mean word length of the sentences used in this phase was 1.62 (*SD* = 0.52). Consistent with the phase 1 result, a larger segmentation disagreement score was associated with longer log-transformed single fixation durations (*b* = 0. 0052, *SE* = 0.0021, *t* = 2.54, *p* < .05; Table 2). Figure 3 (right panel) illustrates the partial relationship between the disagreement score and the log-transformed single fixation durations for the phase 2 data.

**Table 2:** LMM results on log-transformed single fixation durations of phase 2 selected sentences

	Estimates	Standard errors	*t* values
Intercept	2.39	0.0050	482.45*
Word frequency	−0.011	0.0030	−3.75*
Word length	−0.015	0.0025	−5.94*
Word number of strokes	0.015	0.0027	5.80*
Segmentation disagreement	0.0092	0.0018	5.05*

* *p* < .001

The above analyses are based on a converted segmentation score, which considers only the general degree of segmentation (dis)agreement. This converted score does not differentiate whether most participants segmented a character string ABCD into one word or multiple words. As long as there is great consistency, the scores will be the same. For instance, a raw agreement score of 0.1, which indicates high consistency in not assigning a boundary, is considered to have the same degree of (disagreement) as a score of 0.9, which indicates high consistency in assigning a boundary. However, as pointed out by an anonymous reviewer, the string ABCD is perceived as a single long word when the raw agreement score is low, while it is segmented into multiple short words (e.g., AB and CD) when the agreement score is high. Accordingly, the processing may be different in the two cases. Specifically, there might be a general advantage of processing character strings with high segmentation agreement because they represented shorter words. At the same time, character strings with low segmentation agreement should also be processed more easily because of the high level of segmentation consistency (or low level of segmentation ambiguity). In contrast, character strings with mid-range agreement scores were ambiguous in terms of word segmentation, and should be most effortful to process. In other words, an inverted U-shape relationship was expected.

**Fig. 4:**
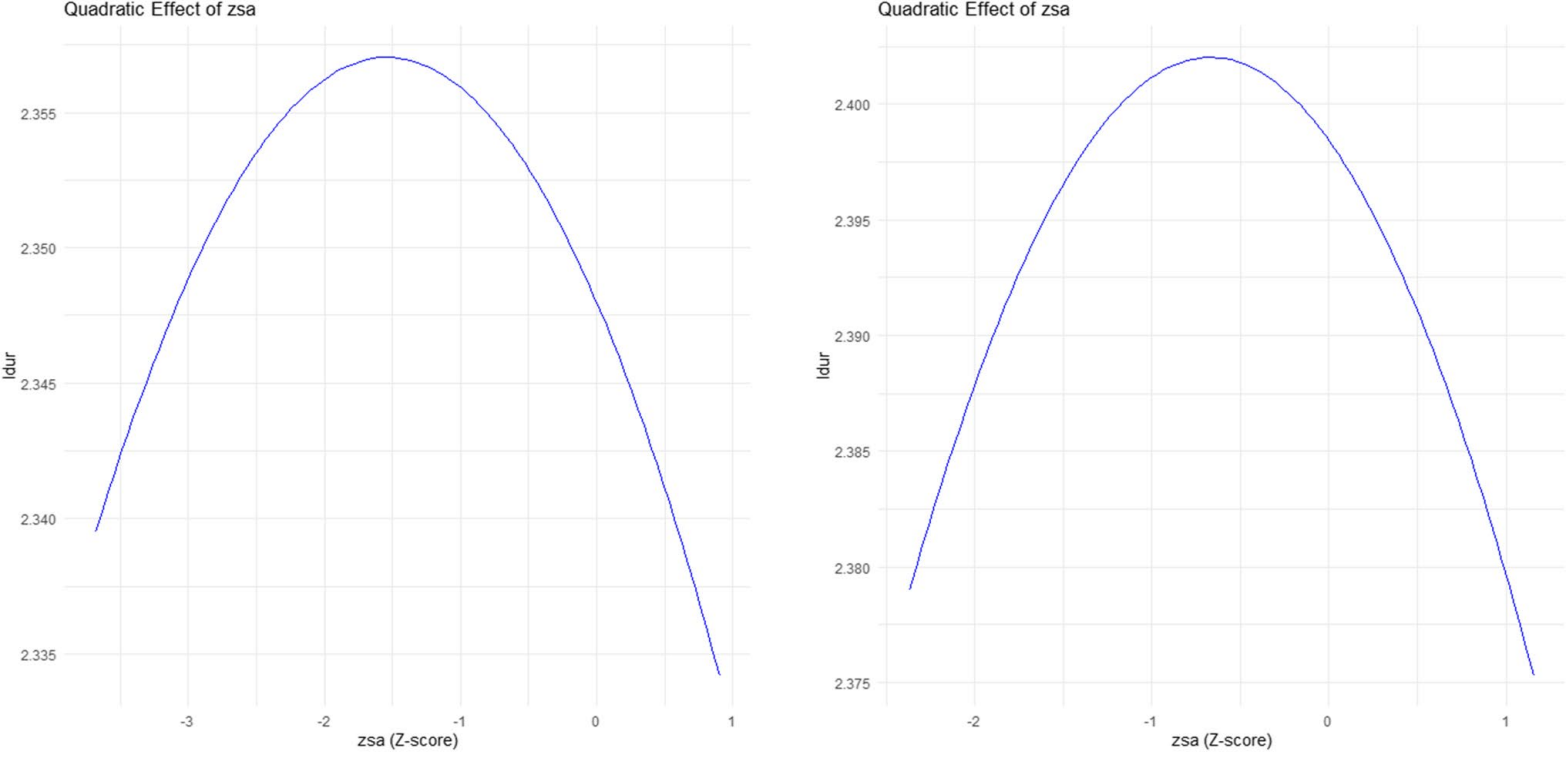
The partial relationship between the quadratic term of z-score transformed segmentation agreements and log-transformed single fixation duration in the BSC data (left panel) and phase 2 data (right panel)

This possibility was tested in two additional models (one for each phase) that examined the relationship between the raw agreement scores and the single fixation times. As in the first set of analyses, the raw agreement scores were logit and *z*-score-transformed. The linear and quadratic effects of the agreement scores were entered into the models. Table 3 shows the results for phase 1 data. After controlling the effects of other variables, both the linear (*b* = −0.012, *SE* = 0.0021, *t* = −5.67, *p* < .001) and the quadratic effects of segmentation agreement (*b* = −0.0038, *SE* = 0.0017, *t* = −2.62, *p* < .01) were statistically significant. Note that the regression coefficients were negative because the predictor was agreement score, which is opposite to the disagreement score used above. The quadratic effect is illustrated in Fig. 4 (left). As expected, fixation durations were longer in the mid-range of segmentation agreement.

**Table 3:** Alternative LMM that tests the linear and quadratic effects of segmentation agreement score on log-transformed single fixation durations of phase 1 sentences

	Estimates	Standard errors	*t* values
Intercept	2.35	0.0076	307.44**
Word frequency	−0.010	0.0024	−3.54**
Word length	−0.0085	0.0022	−4.69**
Predictability	−0.0036	0.0018	−2.01*
Word number of strokes	0.013	0.0020	6.52**
Segmentation agreement (linear)	−0.012	0.0021	−5.67**
Segmentation agreement (quadratic)	−0.0038	0.0015	−2.62*

* *p* < .05; ** *p* < .001

The same analysis was conducted for the phase 2 data. Consistent with the phase 1 results, both the linear (*b* = −0.011, *SE* = 0.0023, *t* = −4.58, *p* < .001) and the quadratic effects of segmentation agreement (*b* = −0.0080, *SE* = 0.0017, *t* = −4.79, *p* < .001) were statistically significant (Table 4). Figure 4 (right) shows the quadratic effect. Again, fixation durations were longer in the mid-range of segmentation agreement.

**Table 4:** Alternative LMM that tests the linear and quadratic effects of segmentation agreement score on log-transformed single fixation durations of phase 2 sentences

	Estimates	Standard errors	*t* values
Intercept	2.40	0.0052	460.80*
Word frequency	−0.012	0.00301	−3.70*
Word length	−0.016	0.0025	−6.13*
Word number of strokes	0.016	0.0027	5.91*
Segmentation agreement (linear)	−0.011	0.0023	−4.58*
Segmentation agreement (quadratic)	−0.0080	0.0017	−4.79*

* *p* < .001

Other attempts in developing comprehensive word segmentation scores are explained in detail in the Appendix at https://osf.io/m3rcf/. In short, different scores have led to highly consistent results on eye movements.

## Discussion

### Segmentation agreement score

In this study, we introduce the CWSA corpus, which comprises word segmentation agreement scores provided by 80 participants for 500 Chinese sentences. These sentences were not selected for having a high degree of word boundary ambiguity. Instead, they were extracted from various sources and intended to represent the typical reading experience of proficient Chinese readers in their daily lives. Indeed, consistent with previous reports (Pan et al., 2022a; Yan et al., 2010), the participants exhibited a remarkably high level of overall segmentation agreement, with a grand average agreement score of 0.92. However, despite the high level of global agreement, we also observed instances of local disagreement. Depending on the strictness of the criterion used, approximately 9% to 15% of the boundaries in the corpus had low agreement scores. Furthermore, a substantial proportion of the sentences (ranging from 85% to 95%) contained at least one ambiguous word boundary.

Further analysis of the ambiguous word boundaries revealed that they could be classified into three main categories. The first category involves characters that function similarly to affixes, such as “了” and “不.” Participants exhibited disagreements regarding whether these characters should be treated as independent one-character words or as part of the whole inflected or derived words. It is important to note that compounding is the primary method for creating morphologically complex words in Chinese, while inflection and derivation are relatively rare (e.g., McBride-Chang et al., 2008). Additionally, ordinary Chinese speakers typically do not receive explicit training in analyzing the morphological structure of Chinese words, as it is not typically included in the Chinese language education curriculum in schools. Therefore, this kind of ambiguity may be attributed to insufficient knowledge or experience with Chinese inflected and derived words.

The second category of ambiguous word boundaries involves two adjacent compound words, such as “科學” and “教育.” These words can be used independently in other contexts and typically do not lead to ambiguity in word segmentation. However, when these words appeared together in a sentence, some participants treated them as separate words, while others tended to combine them into a single long word with a modifier–head structure. In the CWSA corpus, this type of ambiguity is the most prevalent, and similar cases can also be found in English, such as “video game” versus “videogame,” This suggests that the underlying cause of this kind of ambiguity could be a universal phenomenon. It is possible that when a new compound word emerges, language users may not immediately establish its lexical representation. Lexical representation only forms when the new compound is used frequently enough that it enters the common lexicon and achieves consensus among language users. For example, it is believed that “baseball” was originally two separate words “base ball,” and it was once “base-ball” before taking the current single-word form (Bevis, 2006). However, in Chinese, the absence of word boundaries makes such consensus difficult to achieve. As a result, judgments about the lexical status of the new compound word remain idiosyncratic, leading to low segmentation agreement in such cases.

There are also other cases of word boundary ambiguity that cannot be classified into the two main categories and yet are too few and diverse to form a third category. This diversity underscores the basic premise that uncertainty in word segmentation is not limited to some special cases but is a pervasive phenomenon in Chinese (Hoosain, 1992). Interestingly, participants occasionally demonstrated high agreement in segmentation even when the character strings fit the two mentioned categories of boundary ambiguity. For example, the raw segmentation agreement score of “不” was 0.6 for “不穩固–unstable,” while that of “不正確–incorrect” was 0.15, indicating a consensus that the latter should be treated as a single three-character word. Similarly, although “稻米品種–rice varieties” has a modifier–head structure, there was a general agreement to segment it into two separate words, “稻米–rice” and “品種–breed,” with a raw agreement score of 0.75 after the character “米.” The reasons for achieving agreement in these cases are not entirely clear and may involve factors such as frequency of usage and sentence context. These cases illustrate the complexity of word segmentation in Chinese, which cannot be solely determined by a linguistic analysis. They also emphasize the value of an empirical approach, like the one used in developing the CWSA corpus, where segmentation agreement data from ordinary Chinese speakers is collected to provide an alternative to expert judgment and linguistic analysis of word boundaries.

### Effects of eye movement control in reading

To validate the segmentation agreement scores in the CWSA corpus, we tested how these scores affect eye movement in reading. It is important to acknowledge that reading is a complex process that involves not only the processing of fixated words (Kliegl et al., 2006; Rayner, 2009). Words in the prior context are integrated with the currently fixated words. Readers can also process various types of information beyond the currently fixated word (see Kliegl et al., 2006, and Rayner, 2009, for reviews), resulting in parafoveal-on-foveal effects (Kliegl et al., 2007; but see Drieghe et al., 2008, for alternative explanation). However, for the purpose of validation, we adopted a simplified approach and focused on examining the effects of (dis)agreement scores solely on the currently fixation words. This may be the reason why the effects of segmentation (dis)agreement are quite modest. However, the sizes of regression coefficients of the agreement indices are in the same order as the well-established effects of word frequency or word length (see Tables 1, 2, 3 and 4).

In the first set of analyses using the converted disagreement scores, after accounting for the influences of frequency, length, predictability, and visual complexity of the fixated words, an increased disagreement among participants regarding word boundaries was associated with longer single fixation durations. A high level of disagreement in word segmentation likely results in more challenging processing for at least two reasons. Firstly, the ambiguity in word boundaries needs to be resolved before lexical access can occur. Secondly, in the eye movement datasets, character strings with ambiguous boundaries are treated as two separate words. With a high disagreement on these cases, some readers indeed perceive them as single, longer words, which are generally processed more slowly. The increased processing difficulty straightforwardly explains the longer fixation durations observed when disagreement increased.

In the second set of analyses using the agreement scores, an inverted U-shape relationship was found between segmentation agreement and fixation duration. Character strings with boundary ambiguity (i.e., those with mid-range agreement scores) were processed more slowly than those with clear boundaries. At the same time, there was a linear effect of agreement, such that character strings with higher agreement scores were processed faster overall. It might be attributed to the fact that these character strings were perceived as multiple shorter words, and single fixation durations only measured the durations of the first short word.

As mentioned earlier, eye movement behaviors during reading are also influenced by properties of preceding and succeeding words. Given that we have not included these factors in our simplified analyses, we will refrain from further discussing and interpreting the results with regard to specific accounts of word segmentation in Chinese. Instead, we aim to emphasize the validity of the CWSA corpus by showing that the segmentation agreement scores can have significant effects in reading behaviors. While the specific details of the effects remain to be clarified, we believe that the data provided by the CWSA corpus can make a substantial contribution to future studies on this topic.

### Data availability and future directions

The CWSA corpus can be found at https://osf.io/m3rcf/. The CWSA.xlsx file is a Microsoft Excel file that contains two spreadsheets. Firstly, the “global” spreadsheet contains the 500 sentences, together with the raw (range = 0 to 1) and converted (range = 0.5 to 1) segmented agreement scores of each character in each sentence. In this spreadsheet, cells with NA refer to segmentation scores being “not available” because of variation in sentence length. This spreadsheet also includes information about the global mean agreement score and whether each sentence contains at least one ambiguous word boundary. Secondly, the “local” spreadsheet contains all character strings with a converted score between 0.5 and 0.7, which corresponds to a raw segmentation score between 0.3 and 0.7. These character strings are considered to have ambiguous boundaries and are grouped into the morphosyntactic type, modifier–head type, and other type as described above. Note that raw segmentation scores are provided in this spreadsheet because the nature of ambiguity (i.e., inclining towards boundary or non-boundary) may be important for some research questions. In addition to the Excel file, we also provide text and PDF versions for the two spreadsheets to improve accessibility.

The files “segdata_bsc.csv” and “segdata_phase2.csv” are comma-separated value files that store the data used for the phase 1 and phase 2 LMM analyses. Because Chinese words cannot be displayed properly in CSV files, a separate excel file “all_words.xlsx” is used to store the item code, the words for each code, the raw segmentation score, the converted score (that equates low segmentation as having high agreement of “no boundary here”), and the two BVC scores. Interested researchers can easily combine the data using functions like VLOOKUP in Excel. The “output_bsc.txt” and “output_phase2.txt” files contain the R scripts and outputs of the analyses. Details about the random effect structure of the final models that converged successfully can be found here. The Appendix, which contains results of analyses based on the BVC scores, can also be found.

In future studies of word segmentation in Chinese reading, researchers can directly use the sentences in the CWSA corpus to conduct new experiments that answer their research questions. Alternatively, they can construct their own sentences based on the character strings provided in the “local” spreadsheet. For those who need more character strings with ambiguous word boundaries, it is recommended that they can make reference to the categories and examples of ambiguity identified in the CWSA corpus and create similar items accordingly. For instance, they can create other items with a modifier–head structure, as this type of ambiguity is highly prevalent in the CWSA corpus. The resulting items are likely to satisfy the need of high segmentation disagreement. However, in this case, it will be important to conduct pilot testing to ensure that the items created are genuinely ambiguous in word boundaries, as even these types of items occasionally exhibit high segmentation agreements. On the other hand, researchers who want to minimize segmentation ambiguity should avoid these modifier–head strings or use the CWSA sentences with a low level of local ambiguity. Another important direction of future studies is to have the same participants providing word segmentation and reading data simultaneously. This will allow researchers to directly examine how the individual differences in the perceived word boundaries directly affect eye movement behaviors in reading. Indeed, while previous studies (Hoosain, 1992; Liu et al., 2013) and the present report have demonstrated that segmentation ambiguity is common in Chinese, it remains unclear what psychological and linguistic factors would underlie the segmentation process itself. For example, will readers with higher Chinese proficiency tend to have a specific way to segment Chinese words? The CWSA corpus will be a valuable resource in pursing further research to investigate this and other research questions.

## Data Availability

All data and materials are available at https://osf.io/m3rcf/.

## Appendix: Additional analyses based on “boundary certainty values”

In addition to the analyses based on raw segmentation scores, we also developed and tested the effects of two “boundary certainty values” (BCV) that are more theory driven. Specifically, for the character string A/BCD/EF, both the raw and the converted segmentation scores are theory-neutral point values (i.e., considering only the boundary A/B or D/E). In contrast, BCV1 of “BCD” is calculated by averaging the segmentation scores of A/B, (1-B/C), (1-C/D), and D/E. In doing so, the within-word segmentation agreement B/C and C/D will play a role in the calculation, thereby implicitly taking word length into account. This calculation also assumes that segmentation ambiguity of the surrounding words will affect the current word’s segmentation, which is a psychologically possible mechanism. BCV2 differs from BCV1 by ignoring the segmentation score of the preceding word. That is, it equals to the average of (1-B/C), (1-C/D) and D/E. While BCV1 allows carry-over effect of segmentation uncertainty from the preceding word, BCV2 assumes straightly sequential segmentation, such that once the preceding characters are grouped into a word, any uncertainty should have been resolved. Consequently, it only includes segmentation score of the current and next words in the calculation. The two BCVs thus represent different theoretical views about Chinese word segmentation.

Analyses involving these BCV produced qualitatively identical results as the raw segmentation scores. Both the linear effect and the quadratic effect were significant, leading to the same inverted U-shape pattern. The fact that the same results are obtained regardless of the exact segmentation indices used provide support to the robustness of the segmentation information provided by CWSA. Because of the similarity, we do not report details of the BCV analyses here. Interested readers can find the results in the Appendix file at https://osf.io/m3rcf/.

Appendix Tables 5, 6, 7 and 8.

**Table 5:** LMM that tests the linear and quadratic effects of BCV1 on log-transformed single fixation durations of phase 1 sentences

	Estimates	Standard errors	*t* values
Intercept	2.34	0.0075	311.95**
Word frequency	-0.013	0.0029	-4.36**
Word length	-0.0098	0.0024	-4.09**
Predictability	-0.0059	0.0021	-2.87*
Word number of strokes	0.014	0.0021	6.98**
BCV1 (linear)	-0.0076	0.0024	-3.18*
BCV1 (quadratic)	-0.0015	0.0005	-2.97*

* *p* < .05; ** *p* < .001

**Table 6:** LMM that tests the linear and quadratic effects of BCV2 on log-transformed single fixation durations of phase 1 sentences

	Estimates	Standard errors	*t* values
Intercept	2.34	0.0075	311.97*
Word frequency	-0.013	0.0029	-4.29*
Word length	-0.0094	0.0024	-3.91*
Predictability	-0.0040	0.0021	-1.94
Word number of strokes	0.014	0.0021	6.68*
BCV2 (linear)	-0.012	0.0026	-4.70*
BCV2 (quadratic)	-0.0021	0.0005	-4.07*

* *p* < .001

**Table 7:** LMM that tests the linear and quadratic effects of BCV1 on log-transformed single fixation durations of phase 2 sentences

	Estimates	Standard errors	*t* values
Intercept	2.39	0.0052	457.52**
Word frequency	-0.013	0.0026	-5.21**
Word length	-0.0056	0.0032	-1.71
Word number of strokes	0.016	0.0027	5.83**
BCV1 (linear)	-0.0152	0.0031	-4.97**
BCV1 (quadratic)	-0.0044	0.0018	-2.52*

* *p* < .05; ** *p* < .001

**Table 8:** LMM that tests the linear and quadratic effects of BCV2 on log-transformed single fixation durations of phase 2 sentences

	Estimates	Standard errors	*t* values
Intercept	2.40	0.0053	455.00**
Word frequency	-0.014	0.0025	-5.47**
Word length	-0.0073	0.0032	-2.36*
Word number of strokes	0.015	0.0027	5.58**
BCV2 (linear)	-0.022	0.0037	-5.86**
BCV2 (quadratic)	-0.0078	0.0019	-4.06**

* *p* < .05; ** *p* < .001

## Footnotes

1 It should be noted that these translations were unofficial and had not undergone the vigorous back-translation procedure as the official MECO materials, but these sentences increased the diversity of the sentences tested.

## Code availability

The code used for analyses is available at https://osf.io/m3rcf/.
